# WEE1 inhibition in pancreatic cancer cells is dependent on DNA repair status in a context dependent manner

**DOI:** 10.1038/srep33323

**Published:** 2016-09-12

**Authors:** Shruti Lal, Mahsa Zarei, Saswati N. Chand, Emanuela Dylgjeri, Nicole C. Mambelli-Lisboa, Michael J. Pishvaian, Charles J. Yeo, Jordan M. Winter, Jonathan R. Brody

**Affiliations:** 1 Department of Surgery, Division of Surgical Research; Jefferson Pancreas, Biliary and Related Cancer Center; Jefferson Medical College; Thomas Jefferson University, Philadelphia, PA, USA; 2 Lombardi Comprehensive Cancer Center, Georgetown University Medical Center, 3800 Reservoir Road, NW, 20057 Washington DC, USA

## Abstract

Pancreatic ductal adenocarcinoma (PDA) is a lethal disease, in part, because of the lack of effective targeted therapeutic options. MK-1775 (also known as AZD1775), a mitotic inhibitor, has been demonstrated to enhance the anti-tumor effects of DNA damaging agents such as gemcitabine. We evaluated the efficacy of MK-1775 alone or in combination with DNA damaging agents (MMC or oxaliplatin) in PDA cell lines that are either DNA repair proficient (DDR-P) or deficient (DDR-D). PDA cell lines PL11, Hs 766T and Capan-1 harboring naturally selected mutations in DNA repair genes *FANCC*, *FANCG* and *BRCA2* respectively, were less sensitive to MK-1775 as compared to two out of four representative DDR-P (MIA PaCa2 and PANC-1) cell lines. Accordingly, DDR-P cells exhibit reduced sensitivity to MK-1775 upon siRNA silencing of DNA repair genes, *BRCA2* or *FANCD2*, compared to control cells. Only DDR-P cells showed increased apoptosis as a result of early mitotic entry and catastrophe compared to DDR-D cells. Taken together with other recently published reports, our results add another level of evidence that the efficacy of WEE1 inhibition is influenced by the DNA repair status of a cell and may also be dependent on the tumor type and model evaluated.

Pancreatic ductal adenocarcinoma (PDA) is a lethal disease with a dismal 5-year survival rate of only 7% in all those afflicted^1^. Despite the vast genetic knowledge gathered from sequencing PDA genomes, there are no proven current FDA-approved targeted regimens to treat specific genetic variants of PDA^2^. However, a promising off-label ‘targeted’ treatment approach currently under clinical investigation (NCT02042378, NCT02511223) targets PDAs harboring mutations in DNA repair (DDR) genes (such as *BRCA1*, *BRCA2* and *PALB*2) using Poly ADP ribose polymerase (PARP) inhibitors. Small retrospective studies and experience with other cancer types suggest that platinum agents may also be effective against DDR deficient PDAs^3^,^4^,^5^. In fact, ~10% of PDA patients harbor relevant DDR-mutations and may benefit from this molecular tailored therapy^6^.

In the past decade, WEE1 inhibition has been developed as an anti-cancer therapy that targets a cell cycle checkpoint activated in response to DNA damage, and therefore, has been tested as both a single agent therapy and in combination with DNA damaging agents such as gemcitabine, carboplatin and cisplatin^7^,^8^,^9^,^10^,^11^,^12^,^13^,^14^,^15^. WEE1 is a key regulator of cell cycle progression and genomic stability, and is thought to play a critical role in tumors that harbor an impaired G1/S checkpoint related to a non-functional p53 pathway^16^ seen in most cancers^9^,^17^. Consistent with WEE1’s role in tumorigenesis and chemoresistance, it is highly expressed in various cancers including PDA^8^,^10^,^14^,^18^.

Supporting the notion that WEE1 is a good candidate target in PDA cells, we recently demonstrated that WEE1 is rapidly upregulated in PDA upon cytotoxic stress via a post-transcriptional mechanism regulated by the RNA binding protein, HuR^18^. Under DNA damage stress, HuR’s regulation of WEE1 expression activates the G2/M checkpoint through increased CDK1 phosphorylation^18^. This novel stress response mechanism supports a chemoresistant PDA phenotype and demonstrated that rapid translation of WEE1 prevented PDA cells from catastrophic DNA damage^18^,^19^,^20^,^21^,^22^. These data along with previous reports support the hypothesis that bypassing the G2/M checkpoint via WEE1 inhibition in cancer cells should force mitotic entry without adequate DNA repair, and thus result in mitotic catastrophe and apoptosis. In this context, a first in class inhibitor of WEE1, MK-1775 (also known as AZD1775 and chemical structure: 2-allyl-1-(6-(2-hydroxypropan-2-yl)pyridin-2-yl)-6-((4-(4-methylpiperazin-1-yl)phenyl)amino)-1H-pyrazolo[3,4-d]pyrimidin-3(2H)-one) has been explored in a number of pre-clinical and clinical settings including: 1) as a monotherapy in sarcoma cells and refractory solid tumors^11^,^23^; 2) in combination with gemcitabine, cisplatin, 5-fluorouracil and carboplatin in ovarian and colon cancer cells^7^,^8^; 3) in combination with gemcitabine in patient-driven pancreatic mouse xenografts^14^ and a non-selective HDAC inhibitor panobinostat in pancreatic cancer^15^; 4) as a monotherapy in breast cancer^17^; 5) in combination with radiation in glioblastoma^24^ and in current clinical trials in PDA^25^,^26^. Taken together, these studies suggest that WEE1 inhibition can be clinically effective as monotherapy as well as in combination with conventional DNA damaging agents.

Although WEE1 inhibition is a promising therapeutic strategy, pre- selecting tumors to optimize its efficacy is crucial. One of the best defined subgroups tailored for “personalized” therapy are tumors exhibiting “*BRCAness”*, characterized in ~10% of PDAs and suggested to be a conserved molecular signature^27^,^28^,^29^. This select subset includes genetic defects in components of the Fanconi anemia (FA) DNA repair pathway, as seen to occur in the autosomal recessive disorder, FA syndrome. However, germline heterozygous genotypes, or somatic events, may lead to sporadic pancreatic or other cancers^30^,^31^. The FA family of genes is made up of 15 core gene members (FANC A, B, C, D1 (BRCA2), D2, E, F, G, I, J (BRIP1), L, M, N (PALB2), O and P) that have prominent roles in DDR pathways. Seven of the FA proteins (FANC A, B, C, E, F, G and L) form a core complex required for the downstream monoubiquitination of FANCD2 and FANCI^30^,^32^,^33^,^34^,^35^,^36^. A small subset of PDAs harbor mutations (somatic or germline) in *FANCG*, *PALB2* and *FANCC* genes^2^,^37^ with biallelic gene inactivation. Consistent with these findings, FA-defective PDA lines Hs 766T (defective *FANCG*), PL11 (defective *FANCC*) and Capan-1 (defective *BRCA2*) showed hypersensitivity to DNA damaging agents such as mitomycin C (MMC) or cisplatin^35^. Recently, Waddell *et al*. provided a *BRCA* mutational signature of PDA patients associated with platinum drug sensitivity based on whole-genome sequencing^38^. More recently, Kausar *et al*. demonstrated that MK-1775 through WEE1 inhibition sensitized *BRCA2* wild-type (MIA PaCa2 and PANC-1) cells, but not the DDR-deficient (*BRCA2* mutant) PDA cells to gemcitabine-radiation^39^. These data suggest that mutations in the *BRCA* pathway and defects in related DDR genes can enhance the therapeutic intervention for a subset of pancreatic cancer patients^38^.

Building on the emerging enthusiasm to molecularly profile PDA genomes and categorize them according to DNA damage repair capability^38^ along with a recent functional genetic screen identifying FA/homologous repair genes sensitizing genes for WEE1 inhibition^40^, we investigated the efficacy of WEE1 inhibition in the context of DDR status in PDA cells. Results obtained from this study provide compelling evidence that MK-1775 may be less effective in a subset of PDAs harboring somatic *FANCC*, *FANCG* or *BRCA2* mutations.

## Results

### MK-1775 is more effective against DDR-proficient PDA cells compared to DDR-deficient PDA cells

**Figure 1:**
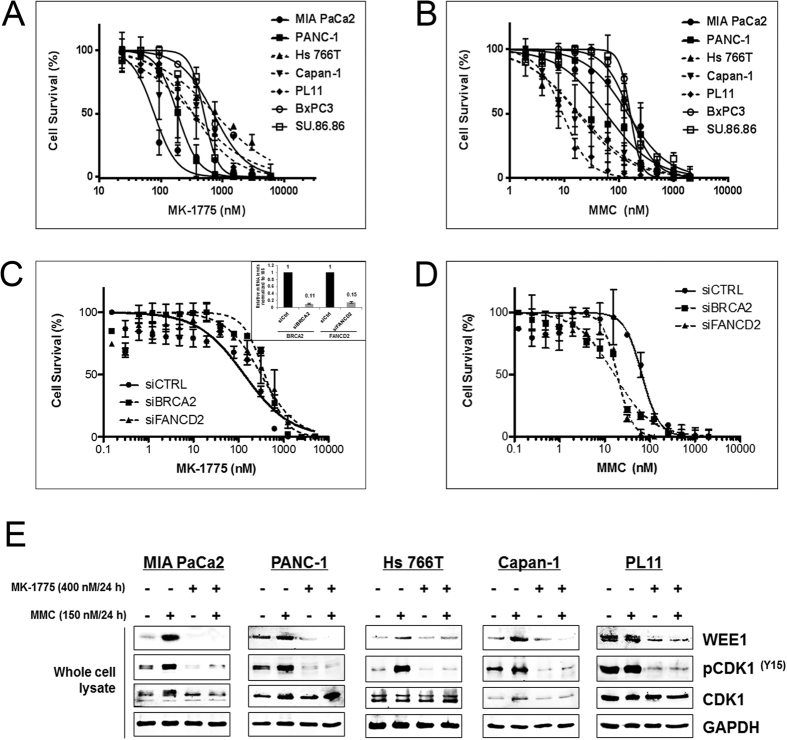
PDA cells with deficiency in DNA repair genes causes resistance to MK-1775 treatment. A short-term (7-days) cell survival assay assessed in MIA PaCa2, PANC-1, SU.86.86 and BxPC3 (*FA*/*BRCA2* proficient), Capan-1 (*BRCA2* deficient), Hs 766T (*FANCG* deficient) and PL11 (*FANCC* deficient) PDA cell lines after treatment with: (**A)** MK-1775 and (**B)** MMC. (**C)** MIA PaCa2 cells were transfected with siRNA oligos against *BRCA2* and *FANCD2*. Quantitative PCR was performed after 48 hours of transfection to confirm the knockdown (inset). A short term (7-days) cell survival assay was performed after treatment with different concentration of MK-1775 and (**D)** MMC. The average of three different experiments was shown in each graph. (**E)** MIA PaCa2, PANC-1, Hs 766T, Capan-1 and PL11 cells were treated with MK-1775 (400 nM/L) and/or MMC (150 nM/L) for 24 hours. Whole cell lysates were prepared using RIPA and western blot was performed to assess the protein expression of WEE1 (90 kDa), pCDK1^(y15)^ (34 kDa), CDK1 (34 kDa) and GAPDH (36 kDa) as loading control. Experiments were validated and performed three times and one of the representative experiments is shown.

To determine the efficacy of MK-1775 in PDA cell lines (MIA PaCa2, PANC-1, PL5, BxPC-3, SU.86.86, Capan-1, Capan-2, PL11 and Hs 766T; Supplementary Fig. S1A, Table 1 and Supplementary Tables S1 and S2), a short-term cell survival assay was performed with increasing concentrations of MK-1775 for 7 days. As a control, a non-transformed pancreatic cell line HPNE was also included in the analysis (Supplementary Fig. S1A). Hs 766T and PL11 cells, defective in *FANCG* and *FANCC* respectively^36^, were less sensitive to MK-1775 compared to the DDR-proficient (DDR-P) cell lines MIA PaCa2 and PANC-1 (Fig. 1A and Table 1). Capan-1 cells, which harbor a *BRCA2* mutation^41^, were more sensitive (2.2 fold change) to MK-1775 compared to Hs 766T cells (Fig. 1A and Table 1), but consistently more resistant (4.3 and 1.8 fold change) compared to the MIA PaCa2 and PANC-1 cell lines, respectively. Surprisingly, HPNE was sensitive to MK-1775 similar to DDR-P cell lines MIA PaCa2 and PANC-1 (Supplementary Fig. S1A and Supplementary Table S1). Of note, SU.86.86 and BxPC3 cells that are DNA repair-proficient were also resistant to MK-1775 (Fig. 1A, Table 1 and Supplementary Table S2). Wang *et al*. also showed that BxPC3 are similar in sensitivity to MK-1775 compared to MIA PaCa2 and PANC-1 cells^15^. To expand on this observation, we then treated SU.86.86 and BxPC3 cells with a constant sub-lethal dose of MMC along with different titrated concentrations of MK-1775 (Supplementary Fig. S1B and Supplementary Table S3) or with a constant sub-lethal dose of MK-1775 along with different titrated concentrations of MMC (Supplementary Fig. S1C and Supplementary Table S3). In line with our overall observations, neither did a pre-treatment with low dose of MMC sensitize SU.86.86 and BxPC3 cells to MK-1775, nor a pre-treatment with a low dose of MK-1775 create a resistant phenotype (Supplementary Fig. S1B,C and Supplementary Table S3). A possible explanation of their resistance could be additional/alternate drug resistance mechanisms or predictive biomarkers other than those carried by MIA PaCa2 and PANC-1 cells (see discussion).

In lieu of the above results, we performed subsequent experiments in five selected, representative PDA cells lines namely MIA PaCa2 and PANC-1 (DNA repair proficient, DDR-P) and Capan-1, Hs 766T and PL11 (DNA repair deficient, DDR-D) (Table 1 and Supplementary Table S2), all of which also have deficient *p53* status. Based on FA biology and the sequence of the signaling cascade, FANCD2 foci are not expected in the *FANCC* (cell line PL11) and *FANCG* (cell line Hs 766T) deficient cells, but should be detectable in FA proficient (MIA PaCa2 and PANC-1) and *BRCA2* deficient cells (Capan-1)^42^. To confirm the integrity of our DDR-deficient PDA lines, all five PDA cell lines were screened for FANCD2 foci formation by immunofluorescence assay (Supplementary Fig. S1D). Additionally, we validated previously published reports that cell lines with defects in the FA pathway are sensitive to inter-strand crosslinking agents such as mitomycin C (MMC)^35^ (Fig. 1B) and oxaliplatin (Supplementary Fig. S1E). Dose response data with MK-1775, MMC and oxaliplatin are summarized in Table 1 and Supplementary Tables S1 and S4. To validate the results obtained in the endogenous repair deficient cell lines, we transiently transfected the DDR-P cells (MIA PaCa2) with siRNA oligos against *FANCD2* and *BRCA2* (Fig. 1C inset). Consistent with the above results, silencing either *FANCD2* or *BRCA2* induced resistance to MK-1775 as compared to control transfected cells (Fig. 1C and Supplementary Table S5). Similar results were obtained in another DDR-P cell line, PL5 cells (Supplementary Fig. S1F and Supplementary Table S5). *FANCD2* or *BRCA2* silencing sensitizes the cells to MMC (Fig. 1D), in agreement with previous studies^35^. Interestingly, despite the phenotypic differences observed in cell survival, all five PDA cell lines respond mechanistically to WEE1 inhibition (through MK-1775 treatment) as evidenced by a decrease in WEE1 protein expression and downstream phosphorylation of CDK1 (Fig. 1E), as also reported by other studies^14^,^43^. These data suggest that endogenous genetic defects occurring in PDA cells influence their sensitivity to MK-1775.

### MK-1775 is more effective against non-pancreatic DDR-deficient cancer cells

To further validate the observed resistance in DDR-D cell lines, we evaluated the efficacy of MK-1775 in isogenic cell culture models of recombinantly modified *FANCC-null* human colon cancer cell line (RKO) and *BRCA2-null* human colon cancer cell line (DLD1) (Supplementary Fig. S2A and Supplementary Table S6)^32^,^44^. As previously published, both *FANCC*- and *BRCA2*-deficient cells were more sensitive to MMC treatment (Supplementary Fig. S2B and Supplementary Table S6)^32^,^44^,^45^. Surprisingly, we observed that BRCA2 (+/−), BRCA2 (−/−) and FANCC (−/−) clones were more sensitive to MK-1775 than parental clones (Supplementary Fig. S2A). Of note, Aarts *et al*. performed a similar assay using recombinantly modified *BRCA2* in DLD1 cell lines and failed to observe any differences in the MK-1775 sensitivity between *BRCA2*-proficient and -deficient cells. Moreover, siRNA- mediated BRCA2 knockdown did not significantly increase MK-1775 sensitivity (cell viability >50%) compared to vehicle treated cells^40^. However, Kausar *et al*. observed that BRCA2 wild-type isogenic cells were sensitive to MK-1775 (AZD-1775) upon gemcitabine-radiation treatment as compared to artificially knocked out BRCA2 isogenic cells^39^. This suggests that MK-1775 sensitivity to cells with genetically disrupted FA genes is both context (e.g., MSI vs CIN) and cell lineage dependent (e.g., colon cancer cells vs. pancreatic cancer cells)^23^.

### MK-1775 monotherapy induces apoptotic cell death in DDR-proficient pancreatic cancer cells

**Figure 2:**
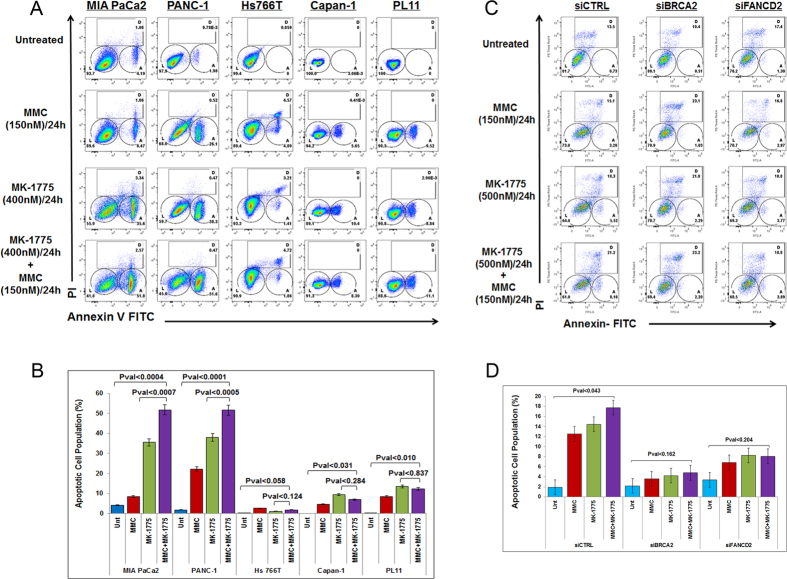
MK-1775 induces cell death in PDA cells with deficiency in DNA repair genes. (**A)** MIA PaCa2, PANC-1, Hs 766T, Capan-1 and PL11 cells were treated with MMC (150 nM/L) and MK-1775 (400 nM/L) for 24 hours and subjected to Annexin V/PI staining to quantify percentage of apoptotic (V+/PI−) cells. (**B)** The average quantification of three replicates shown in (**A**). (**C)** Annexin V/PI positive cells were quantified in MIA PaCa2 cells after transfection with *siBRCA2* and *siFANCD2*. (**D)** The average quantification of two replicates shown in (**C**). L: Live cells; A: Apoptotic cells; D: Dead cells.

Apoptotic cell death in response to MK-1775 treatment was evaluated by Annexin V-PI (Supplementary Fig. S2C) in both DDR-P and DDR-D cell lines. Since all five cell lines exert different IC_50_, we used a single dose of MK-1775 (400 nM) closer to the IC_50_ of DDR-D cell lines to treat the cells. Our results indicated that the DDR-P cell lines MIA PaCa2 and PANC-1 showed apoptotic cell death by MMC (150 nM^18^; 24 h) and MK-1775 (400 nM; 24 h), as well as with combined therapies (Fig. 2A,B). However, *FANCG*-deficient Hs 766T cells, *FANCC*-deficient PL11, and *BRCA2*-deficient Capan-1 cells did not exert the same effect when treated with MK-1775 (Fig. 2A,B). Importantly, co-treatment with MMC also failed to enhance cell death in DDR-D cells (Fig. 2A,B and Supplementary Table S7). We further validated these results in siRNA *FANCD2* and/or *BRCA2* transfected DDR-P MIA PaCa2 cell lines (Supplementary Fig. S2D). In the control transfected (si-scrambled- control) cells, MK-1775 treatment alone as well as in combination with MMC induced significant cell death compared to untreated cells (Fig. 2C,D). Conversely, cells transfected with *siFANCD2* or *siBRCA2* failed to undergo apoptotic cell death after treatment with MK-1775 or combination treatment with MMC (Fig. 2C,D). Since, HPNE cells (a non-PDA cell line) exert sensitivity to MK-1775 (Supplementary Fig. S1A), we also performed apoptotic assay in HPNE cells and observed no significant cell death (Supplementary Fig. S2E and Supplementary Table S7). Unlike the observations from short-term cell survival assay, both BxPC3 and SU.86.86 cell lines exert significant cell death when treated in combination with MK-1775 and MMC compared to untreated (Supplementary Fig. S2E and Supplementary Table S7) suggesting that there were indeed more apoptotic cells in these DDR-P cell lines. Taken together, these data demonstrate that MK-1775 induces more cell death in FA pathway proficient cells as compared to DDR-D cells.

### MK-1775 treatment induces abnormal nuclear morphology and replication stress in DDR-proficient pancreatic cancer cells

**Figure 3:**
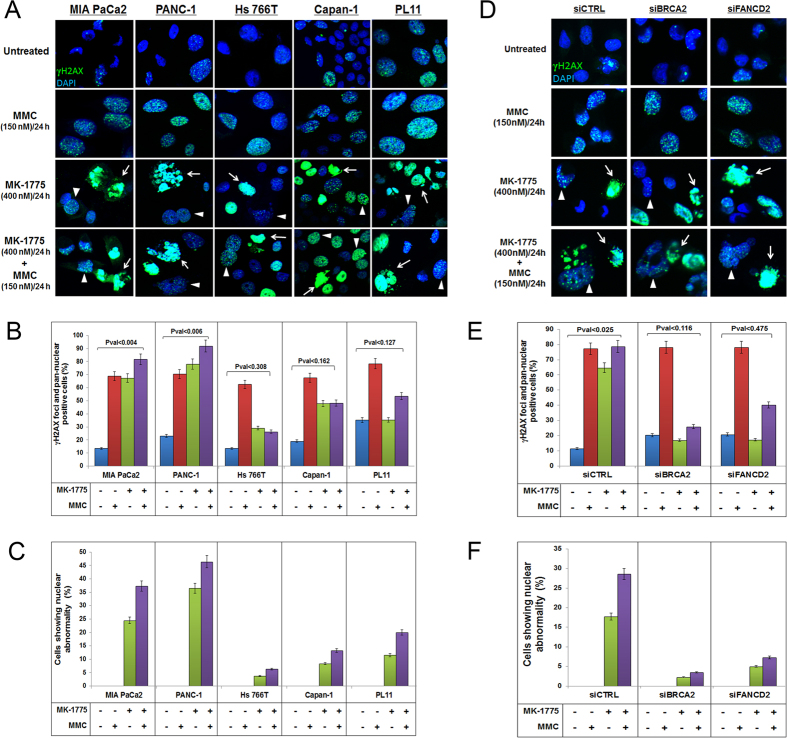
MK-1775 treatment induces abnormal nuclear morphology in PDA cells proficient in DNA repair genes. (**A)** MIA PaCa2, PANC-1, Hs 766T, Capan-1 and PL11 cells were treated with MMC (150 nM/L) and MK-1775 (400 nM/L) for 24 hours and the DNA damage and multi-nucleated phenotype were observed using staining with γH2AX and DAPI. (**B)** Graph showing γH2AX and pan-nuclear positive stained MIA PaCa2, PANC-1, Hs 766T, Capan-1 and PL11 cells. (**C)** Percentage of MIA PaCa2, PANC-1, Hs 766T, Capan-1 and PL11 cells showing nuclear abnormal phenotype. (**D)** MIA PaCa2 cells were transfected with siRNA oligos against *BRCA2* and *FANCD2*; 48 hours later cells were treated with MMC (150 nM/L) and MK-1775 (400 nM/L) for 24 hours and γH2AX foci formation and multi-nucleation was observed. (**E)** γH2AX foci and pan-nuclear staining was assessed after transfections. (**F)** Percentage of transfected cells showing nuclear abnormality. White arrows indicate nuclear abnormality or pan-nuclear staining and arrowhead indicates γH2AX foci formation or DNA damage.

To further evaluate the mechanism of action of MK-1775, all five of our selected cell lines (MIA PaCa2, PANC-1, Hs 766T, Capan-1 and PL11) were either treated with MMC or MK-1775 alone or in combination for 24 hours before immunofluorescence was performed. MMC treatment induced γH2AX foci formation in all cell lines (Fig. 3A,B). However, WEE1 inhibition through MK-1775 treatment induced pan-nuclear γH2AX staining without visible foci consistent with the previous findings (Fig. 3A,B)^46^. Interestingly, MK-1775 treatment caused significant nuclear abnormalities forming multi-nucleated phenotype in DDR-P MIA PaCa-2 and PANC-1 cells suggesting that the cells undergo mitotic catastrophe which was not very evident in the DDR-D cell lines (Fig. 3A,C). We further validated the results by transfecting MIA PaCa2 cells with siRNA oligos against *FANCD2* or *BRCA2*. Results demonstrated that control transfected cells showed more abnormal nuclear morphology, γH2AX foci and pan-nuclear γH2AX staining after treatment with MK-1775 than *siFANCD2* or *BRCA2* transfected cells (Fig. 3D–F). These results suggest that WEE1 inhibition with MK-1775 alone results in a higher degree of replication stress and induced DNA damage in DDR-P cells as compared to DDR-D cells, which is further enhanced upon co-treatment with MMC.

### MK-1775 induces mitotic entry in DDR-proficient pancreatic cancer cells

**Figure 4:**
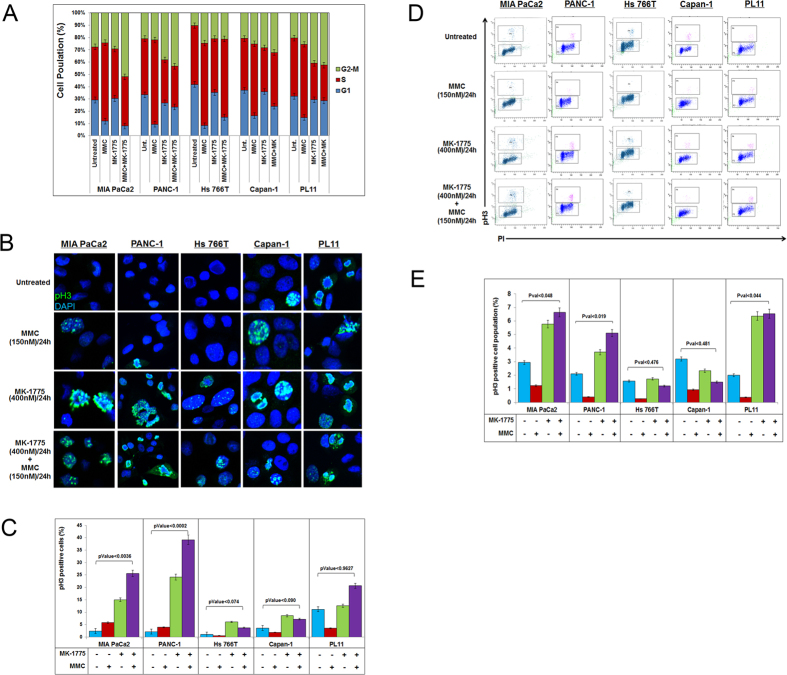
MK-1775 treatment induces mitotic entry and catastrophe in PDA cells proficient in DNA repair genes. (**A)** Cell cycle kinetics was assessed in MIA PaCa2, PANC-1, Hs 766T, Capan-1 and PL11 cells after treatments with MMC (150 nM/L) and MK-1775 (400 nM/L) for 24 hours. Percentage distribution of cells in G1, S and G2/M phases are shown. (**B)** MIA PaCa2, PANC-1, Hs 766T, Capan-1 and PL11 cells were treated and stained with pH3 to observe mitotic entry. (**C)** Quantification of pH3 positive cells after treatments shown in (**B**). (**D)** FACS analysis was performed to determine the percentage of pH3 positive cells upon treatments. (**E)** Quantification of pH3 positive cells after treatments shown in (**D**).

It was previously published that WEE1 inhibition via MK-1775 abrogates G2/M cell cycle arrest and enhances early mitotic entry^43^. To further validate this mechanism of action in PDA cells, we performed cell cycle kinetics by pulse-labeling the cells with bromodeoxyuridine (BrdU) followed by MK-1775 and/or MMC treatment for 24 hours. The cell cycle distribution was monitored by the progression of the BrdU-labeled cells. Our results demonstrate that DDR-P cells showed a higher percentage of cells arrested in the G2/M phase of the cell-cycle than DDR-D cells after combination treatment of MK-1775 and MMC, with the exception of PL11 cells that induce a higher percentage of cells in G2/M phase (Fig. 4A). We further determined the mitotic index of cells by immunofluorescence assay. All cell lines were either treated with MMC or MK-1775 alone or in combination for 24 hours and then stained with phospho-histone 3 (pH3), a marker of mitotic entry. Results showed that DDR-P cells induce mitotic entry upon MK-1775 exposure which was further enhanced by combination treatment compared to control cells (Fig. 4B,C). Interestingly, DDR-P PANC-1 cells after combinational treatment showed a higher percentage of cells undergoing mitosis than MK-1775 alone treatment suggesting that there was indeed a significant difference in cell-cycle kinetics which was not evident in BrdU experiment (Fig. 4A,C). In comparison, DDR-D Hs 766T and Capan-1 cells did not show enhanced mitotic entry after MK-1775 alone or in combination with MMC treatments (Fig. 4B,C). However, though DDR-D PL11 cells showed significant mitotic entry compared to Hs 766T and Capan-1 cells, but not significant when compared to untreated control cells (Fig. 4B,C).

We further analyzed the cell cycle kinetics after treatments using pH3 antibody by FACS. Consistent with previous findings, we observed a higher percentage of pH3 positive cells in DDR-P MIA PaCa2 and PANC-1 cells upon combination treatment compared to DDR-D Hs 766T and Capan-1 cells (Fig. 4D,E). Surprisingly, we observed more cells arrested in mitosis in *FANCC*-deficient PL11 cells in response MK-1775 alone or combination treatments (Fig. 4D,E). However, an apoptotic assay indicated less cell death in PL11 cells upon MK-1775 alone or combination treatment compared to DDR-P cells (Fig. 2A). This suggests that PL11 cells were arrested in G2/M phase but did not undergo mitotic catastrophe as observed in DDR-P MIA PaCa2 and PANC-1 cells. A similar trend was observed in all cell lines with oxaliplatin alone or in combination with MK-1775 treatments (Supplementary Fig. S3A–D). Taken together with the cell cycle analyses, an FA-repair deficiency in PDA cells is likely to cause G2/M arrest even with MK-1775 inhibition.

### WEE1 inhibition in combination with a DNA damaging agent induces caspase-dependent cell death in DDR-proficient pancreatic cancer cell lines

**Figure 5:**
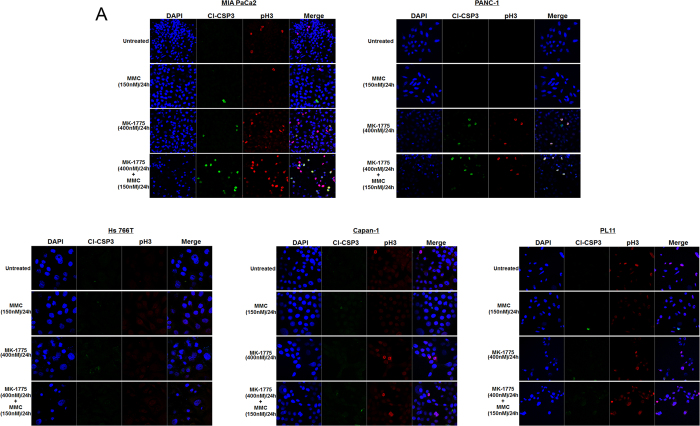
MK-1775 treatment induces mitotic cell death in PDA. (**A)** MIA PaCa2, PANC-1, Hs 766T, Capan-1 and PL11 cells were treated and dual stained with pH3 to observe mitotic entry; and cleaved caspase 3 (CL-CSP3) to observe caspase 3 activity.

To further evaluate that WEE1 inhibition by MK-1775 induces mitotic cell death in DDR-P cells, we conducted immunofluorescence (IF) experiments using pH3 and cleaved caspase 3 (CSP3) to simultaneously assess mitotic entry and cell death, respectively. Our results demonstrate that upon MK-1775 and combination treatments, DDR-P cells (MIA PaCa2 and PANC-1) that stained positive for CSP3 also co-localize with pH3 suggesting that cells are undergoing mitotic catastrophe (Fig. 5A). On the contrary, DDR-D Capan-1 and PL11 cell lines demonstrated mitotic entry as indicated by the increased expression of pH3 upon MK-1775 or combination treatment (Fig. 5A). However, Capan-1 and PL11 cells stained negative for CSP3 suggesting that cells failed to undergo apoptosis (Fig. 5A). As expected, Hs 766T cells were negative for both CSP3 and pH3 staining indicating that MK-1775 failed to induce mitotic entry or cell death (Fig. 5A). These results indicate that MK-1775 in combination with MMC enhances mitotic entry regardless of enormous DNA damage induced by MMC and promotes cell death in DDR-P cells. Whereas, DDR-D (Hs 766T, Capan-1 and PL11) cells demonstrate induction of DNA damage upon MMC treatment (Fig. 3A,B) but MK-1775 failed to induce significant mitotic entry (Fig. 4B–E) or cell death (Fig. 2A,B and Fig. 5) compared to DDR-P cells suggesting that these cells are arrested in the G2/M phase of cell cycle.

## Discussion

Several studies have been conducted to design therapeutic strategies incorporating MK-1775 either as a monotherapy or in combination with chemotherapeutic drugs in different cancers^7^,^8^,^10^,^14^,^23^. The G1/S checkpoint is functionally inactive in most of the cancers mainly because of mutation in *TP53* gene, therefore abrogating the G2/M checkpoint via WEE1 inhibition followed by DNA damage offers a promising therapeutic opportunity to kill cancer cells. There is compelling evidence to support this strategy that MK-1775 induces forced mitotic entry and combination treatment with DNA damaging agents or radiation promotes apoptosis and reduced tumor growth^10^,^12^. Targeting WEE1 in an effort to enhance therapy in PDA cells is especially thought provoking in light of our recent study that DNA damaging agents can rapidly and potently induce an HuR-dependent WEE1 upregulation^18^.

In this report, we validated previous work that MK-1775 has a cytotoxic effect in a panel of pancreatic cancer cell lines (Supplementary Fig. S1A), which of note, is a different panel of cell lines used in a recent study to define genes involved in WEE1 inhibition (Table 2)^40^. Complementary to our work, Kausar *et al*. concluded that MK-1775 can sensitize HR-proficient PDA cells to gemcitabine chemoradiation and they propose that PDA tumors without underlying HR-defects would respond best to this combination strategy^39^. Expanding on this line of investigation, we previously found that upregulation of WEE1 in PDA cells via HuR occurred in a variety of PDA lines with DDR-proficient and deficient genetic backgrounds^18^. Our results showed that MIA PaCa2 and PANC-1 cells (DDR-P) were significantly more sensitive to MK-1775 treatment compared to Hs 766T, PL11 and Capan-1 pancreatic cancer cell lines (DDR-D) (Fig. 1A). Whereas drug efficacy, as measured by a cell survival assay, depends on DDR function; on a molecular level, PDA cells are not dependent on hallmark DDR functions (Fig. 1A,E). We do note that in our extensive evaluation of a diverse genotype of PDA cell lines (n = 9), this association between DDR-D lines and MK-1775 did not correlate 100% (e.g., BxPC3 and SU.86.86 cells) (Supplementary Table S2). Limitations to making a connection between DDR status and MK-1775 in these cell lines and in all PDA tumors may be related to several factors intrinsic to PDA biology (i.e., other confounding molecular alterations such as PKMYT1 expression which phosphorylates and inhibits WEE1 targets such as CDK1^47^, along with potential unknown drug transporter/metabolism deficiencies within these cell lines).

Contrary to recent publications^23^,^40^, we observed that genetically modified colon cancer cell lines for *BRCA2* and *FANCC* were more sensitive to MK-1775 (Supplementary Fig. S2A). Aarts *et al*. did not observe any such difference in the MK-1775 sensitivity in their genetically modified colon cancer cell lines for BRCA2- proficient and -deficient cells^40^. While Kausar *et al*. also reported that BRCA2-null isogenic colon cell lines were not further sensitized to gemcitabine-radiation by MK-1775 while the complementary wild-type BRCA2 cells were significantly more sensitive (Table 2)^39^. Previously, it was demonstrated that *TP53* interacts with *FANCC* to regulate apoptosis and tumorigenesis upon MMC or radiation exposure in FANCC-deficient cells^48^. However, Rosselli *et al*.^49^ reported reduced- while Kruyt *et al*.^50^ and Ridet *et al*.^51^ showed normal- induction of *TP53* in FA cells. All pancreatic DDR-D cell lines (Hs 766T, PL11 and Capan-1) used in this study are *TP53*-deficient and showed less sensitivity to MK-1775 compared to DDR-P (MIA PaCa2 and PANC-1) cells. However, targeted disruption by homologous recombination RKO and DLD1 cells, which are *TP53*-proficient, were sensitive to MK-1775 compared to parental cell lines (Supplementary Fig. S2A,B and Supplementary Table S6). Moreover, transfecting the RKO and DLD1 cells with siRNA oligos against *TP53* did not alter the MK-1775 efficacy in these cells (data not shown). These data suggest that MK-1775 sensitivity to cells with genetic disruption of DDR genes in both pancreatic and colon cancer cell lines could be context dependent (Table 2). Additional differences between this study and the recently reported findings are that: 1) we evaluated MK-1775 efficacy in naturally disrupted *FANCC* or *BRCA2* cell lines, and 2) different identified FA genes were validated in each instance (Table 2)^23^,^40^. Importantly, a recent report of a Phase I study in patients with refractory solid tumors included 6 *BRCA*-mutated patients^23^. Two of the 6 patients had partial responses (an ovarian and a head and neck tumor). Only one patient with pancreatic cancer was in this subgroup, but withdrew to go on standard-of-care treatment. These data perhaps support the notion that WEE1 inhibition targeted against BRCAness may be context dependent. We certainly do not dispute the data from other laboratories (Table 2)^23^, in fact, we hope that future studies may help determine which tumor systems that harbor BRCA/FA-homologous repair gene defects may cause resistance or sensitivity to WEE1 inhibition.

Although a trend towards decreased sensitivity to WEE1 inhibition in DDR-D cells is apparent from this study, the mechanism of resistance is unclear. One possible explanation of this resistance mechanism would be that upon MK-1775 treatment, alternative checkpoints are activated in DDR-D cell lines, compensating for G2/M checkpoint inactivation and allowing cells to go under efficient DNA repair. Apoptotic assays suggest that MK-1775 did not synergize with MMC to promote cell death in DDR-D cells (Fig. 2A). In addition, cell cycle kinetics also suggests that cells are primarily arrested in G2 phase of the cell cycle in these cells and failed to induce mitotic entry either alone or in combination with DNA damaging agents MMC or oxaliplatin (Fig. 4 and Supplementary Fig. S3). It seems perplexing that there is a decrease in γH2AX positive cells in DDR-D cell lines suggests that these cell lines adapt an alternative G2/M checkpoint mechanism upon MK-1775 treatment to repair the damage. Incorporating data from the drug sensitivity assays, apoptosis and immunofluorescence with cleaved caspase 3 assays, and with the exception of PL11 cells that showed increased mitotic entry with FACS assay (Figs 1, 2 and 5), these findings indicate that DDR-D cells are taking an alternative route to activate G2/M checkpoint. Therefore, it would be more informative to test the efficacy of MK-1775 when combined with other G2/M checkpoint inhibitors. For instance, recent studies also showed that combined inhibition of checkpoint kinase 1 (CHK1), another inducer of G2/M phase arrest, and WEE1 increased therapeutic efficacy and reduced tumor growth. Wang *et al*. also reported that the CHK1 selective inhibitor (LY2603618) enhanced MK-1775 sensitivity in pancreatic cancer cell lines^15^. Mak *et al*. demonstrated that inhibiting more than one of the components of the ATR-CHK1-WEE1 pathway can overcome the pharmacological limitation of these inhibitors^52^, therefore supporting a potentially efficacious strategy to combine MK-1775 and CHK1 inhibitor in DDR-deficient PDA cells.

In summary, our *in vitro* data support the notion that WEE1 inhibition alone may not provide a clinical advantage for PDA patients with mutations specifically in *FANCC*, *FANCG*, and *BRCA2*. We were able to validate these findings by silencing FANCD2 and BRCA2 in DDR-proficient PDA cells. These pre-clinical *in vitro* studies provide a rationale to select tumors based on DDR- gene proficiency, and assess efficacy of WEE1 inhibition in patients according to their specific mutational status. Although this work ultimately needs to be supported by either retrospective analysis of a clinical trial and/or a prospective, biomarker driven clinical trial; for now, our data suggest that molecularly identified DDR-deficient PDAs (i.e., *FANCC*, *FANCG*, and *BRCA2*) should not be treated with MK-1775-based therapies. Additionally, this work adds to the literature that WEE1 inhibition may be context dependent and this line of investigation may guide the selection and timing of other therapies to be used in combination with MK-1775.

## Methods

### Cell culture, transfections and treatments

All pancreatic cancer cells were purchased from ATCC. MIA PaCa2, PANC-1, Hs 766T, HPNE, and Capan-1 cells were cultured in DMEM (Gibco/Invitrogen, Carlsbad, CA); BxPC3, Capan-2, PL11 and SU.86.86 cells were cultured in RPMI 1640. All cells were supplemented with 10% FBS (Gibco/Invitrogen) expect PL11 (15% FBS and insulin), 1% L-glutamine (Gibco/Invitrogen), and 1% penicillin-streptomycin (Invitrogen) at 37 °C in 5% humidified CO_2_ incubators.

For transient transfections, siRNAs against *FANCD2*, *BRCA2* and control oligos (Dharmacon) were transfected using lipofectamine 2000 (Gibco/Invitrogen) as previously described^18^. All cells were harvested 48 hours post-transfection.

All cells were treated with the IC_50_ values of the DNA damaging agent MMC (mitomycin C; (Sigma, St. Louis, MO) and oxaliplatin (Sigma) as previously described^18^ by adding directly into the culture medium. MK-1775 was purchased from Selleckchem, Houston, TX.

### Whole cell Extracts and SDS-PAGE/Western Blotting

Whole cell lysates were prepared using RIPA lysis buffer (Invitrogen) supplemented with phosphatase inhibitor (Pierce; ThermoScientific) by incubating on ice for 10 min followed by centrifugation at 13,000 *g for 15 min at 4 °C as previously described^18^. Samples were mixed 4:1 with 5X Laemmli buffer, boiled for 5 min. Approximately, 30–50 μg of protein were separated using a 10–15% Bis-Tris polyacrylamide gel and transferred to PVDF membrane (Invitrogen). The membrane was blocked in 1:1 Licor Odyssey blocking buffer and incubated with GAPDH (1:1,000, Cell Signaling), pCDK1-Y15 (1:1,000, Cell Signaling), CDK1 (1:1,000, Cell Signaling) or WEE1 (1:1,000, Cell Signaling) antibodies. Protein complexes were visualized with Licor Odyssey imaging system.

### Immunofluorescence

Approximately, 50,000 cells per well were plated on coverslips in 24-well plate. After treatments, cells were washed 2 times with PBS, fixed with 4% paraformaldehyde, permeabilized with 0.2% Triton-X, blocked with 5% goat serum, and incubated with pH3, CSP3 or γH2AX antibodies, as previously described^18^. Cell nuclei were stained with DAPI and coverslips were mounted with DAPI ProLong Gold Antifade (Invitrogen) for analysis with a Zeiss LSM-510 Confocal Laser Microscope. Approximately, 300–500 cells were used for counting the number of pH3 and γH2AX positive nuclei using ImageJ 1.47a software (NIH, Bethesda, MD, USA; http://imagej.nih.gov/ij/).

### FACS for pH3 positive cells

Cells were seeded at 10^6^ cell density in 10 cm dishes and treated with MMC (150 nM/L) and MK-1775 (400 nM/L) for 24 hours. Cells were harvested using cellstripper (Corning), fixed in ice-cold 70% ethanol for 2 hours, permeabilized with 0.25% Triton-X on ice for 10 minutes, incubated with anti-phospho (Ser10)-Histone H3 Ab (Clone 3H10) antibody (1:1,000, Cell Signaling) for 1-2 hours at 4 °C followed by Alexa488-conjugated antibody (1:2,000) for 1 hour in dark at 4 °C, washed and re-suspended in 20 μg/ml propidium iodide supplemented with 100 μg/ml RNase for 15 minutes at RT in the dark. Samples were analyzed on flow cytometry BD LSRII (BD Biosciences, San Jose, CA). 10,000 events were recorded for each sample. Each cell line has its own compensation controls and gating was done according to each cell line unstained/untreated sample. Data was analyzed in FlowJo (FlowJo LLC.).

### Drug Sensitivity and Apoptosis Assays

Cells were seeded at 1,000 cells per well in 96-well plates in triplicates and treated after 24 hours. After 5–7 days of treatment (only 1 dose exposed to cells after the cells are plated for 24 h), cells were washed twice with PBS and lysed with deionized-water for 1 hour at 37 °C. Cells were stained with PicoGreen (Invitrogen), a fluorescent dye that selectively binds double stranded DNA, for 2 hours in dark at RT, as previously described^18^. The intensity of the fluorescent signal correlates to the number of viable or surviving cells. Results were analyzed using GraphPad Prism (GraphPad Software Inc.), La Jolla, CA.

For apoptoic assay, cells were seeded at 10^6^ cell density in 10 cm dishes and treated with MMC (150 nM/L) and/or MK-1775 (400 nM/L) for 24 hours. Annexin V13242 labeling kit (Invitrogen) was used following manufacturer’s protocol to measure the apoptotic cells. Samples were analyzed on flow cytometry BD LSRII (BD Biosciences, San Jose, CA) and 50,000 events were recorded for each sample in Fig. 2A and 10,000 events were recorded for each sample in Fig. 2C. Each cell line has its own compensation controls and gating was done according to each cell line unstained/untreated sample. Gating strategy is shown in Supplementary Fig. S2C. Only V+/PI− cells are considered as apoptotic cells and plotted in the graph. Data was analyzed in FlowJo (FlowJo LLC.).

### Statistical Analysis

All p-values were calculated in GraphPad using paired T-Test function.

## Additional Information

**How to cite this article**: Lal, S. *et al*. WEE1 inhibition in pancreatic cancer cells is dependent on DNA repair status in a context dependent manner. *Sci. Rep*. **6**, 33323; doi: 10.1038/srep33323 (2016).

## Supplementary Material

Supplementary Information

## Figures and Tables

**Table 1:** Table showing IC_50_ values (average of 3 replicates) of MK-1775 and MMC in pancreatic cancer cell lines (shown in Fig. 1A,B).

Cell Lines	FA/BRCA pathway status	BRCA2 Status	p53 Status	MK-1775 IC_50_ (nM;SE)	MMC IC_50_ (nM;SE)	
MIA PaCa2	Proficient	BRCA2 +/+	p53 mutant	77.93 ± 0.039	144.5 ± 0.024	
PANC-1	Proficient	BRCA2 +/+	p53 mutant	185.9 ± 0.037	57.9 ± 0.042	
Hs 766T	Deficient	BRCA2 +/+	p53 mutant	744.5 ± 0.070	19.0 ± 0.059	
Capan-1	Deficient	BRCA2 −/−	p53 mutant	336.0 ± 0.100	17.6 ± 0.044	
PL11	Deficient	BRCA2 +/+	p53 mutant	334.5 ± 0.067	9.3 ± 0.031	
BxPC3	Proficient	BRCA2 +/+	p53 mutant	674.9 ± 0.033	161.3 ± 0.014	
SU.86.86	Proficient	BRCA2 +/+	p53 mutant	500.2 ± 0.050	158.7 ± 0.038	

**Table 2:** Table showing *in-vitro* or clinical studies relating efficacy of MK-1775 and the BRCA2/FA pathway^23^,^39^,^40^.

Study/Author	Year	Tumor Type	Genes Involved	Model	Sensitive/Resistance	Therapy (Mono or Combination)	Pubmed ID
Aarts *et al*.	2015	Colorectal adenocarcinoma	Fanconi anemia: FANCM, BRIP1, FANCE and PALB2 Homologous recombination repair: RAD54B, RECQL4, RAD50, RAD52, BRCA1, BRCA2	*In-vitro*	Sensitive	Mono	25673822
Do *et al*.	2015	Solid Tumor	Homologous recombination repair: BRCA	Clinical Trial	Sensitive	Mono	25964244
Kausar *et al*.	2015	Pancreatic adenocarcinoma	Homologous recombination repair: BRCA	*In-vitro In-vivo*	Resistance	Mono and combination	26585231
Lal *et al*.	2016	Pancreatic adenocarcinoma	Fanconi anemia: FANCC, FANCG Homologous recombination repair: BRCA2	*In-vitro*	Resistance	Mono and combination	Current Study
