# Supplementary material for: WEE1 inhibition in pancreatic cancer cells is dependent on DNA repair status in a context dependent manner
Source: Sci Rep. 2016 Sep 12;6:33323. doi: 10.1038/srep33323 (PMC5018859; doi:10.1038/srep33323)
Supplement: Supplementary Information [file srep33323-s1.pdf]

WEE1 inhibition in pancreatic cancer cells is dependent on DNA repair status in a context dependent manner

Shruti Lal[1], Mahsa Zarei[1], Saswati N. Chand[1], Emanuela Dylgjeri[1], Nicole C. Mambelli-Lisboa[1], Michael J. Pishvaian[2], Charles J. Yeo[1], Jordan M. Winter[1], Jonathan R. Brody[1]

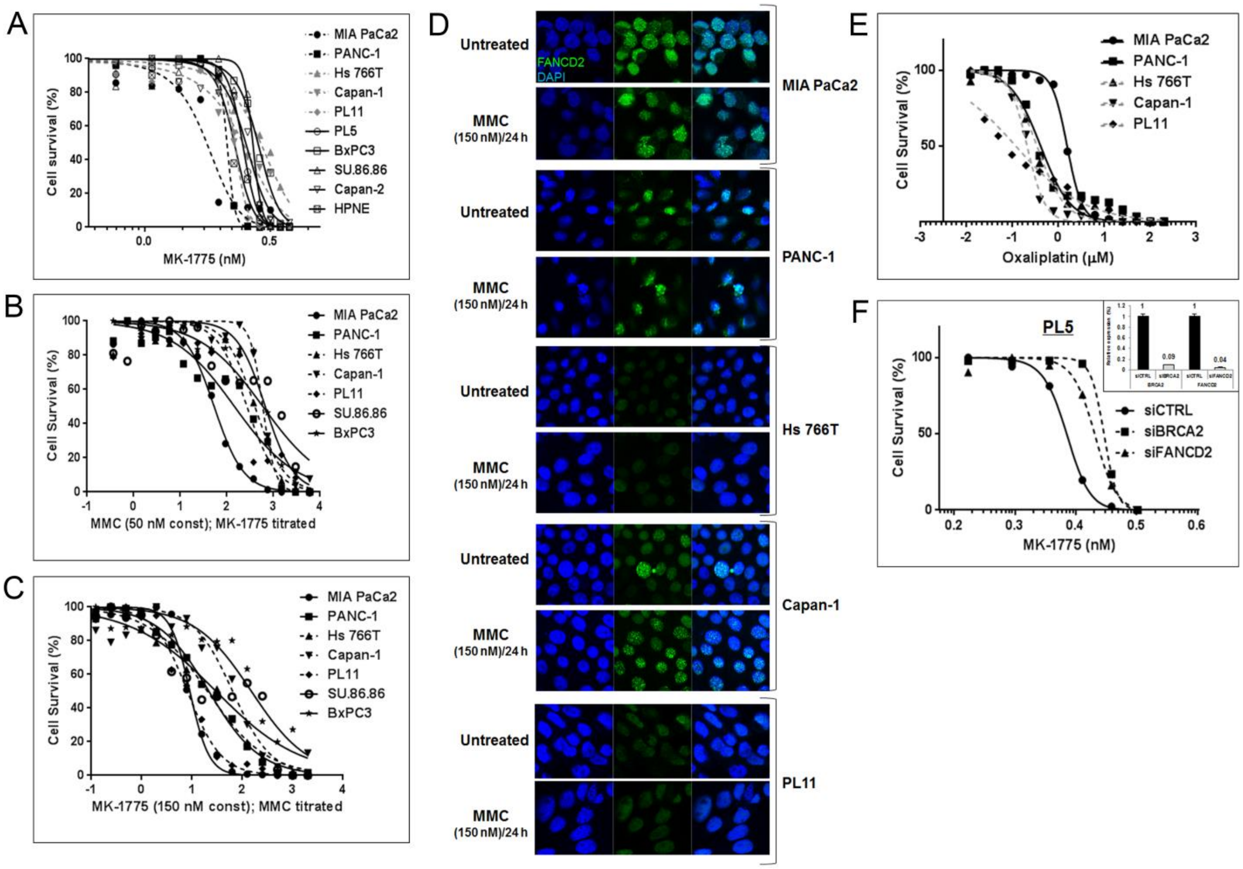

Figure S1: A) PDA and non-PDA HPNE cells were treated with different concentrations of MK-1775 and cell viability was assessed after 7 days. **B)** PDA cells were treated with constant 50 nM concentration of MMC and with different concentration of MK-1775. **C)** PDA cells were treated with constant 150 nM concentration of MK-1775 and with different concentration of MMC. **D)** Immunofluorescence assay to asses FANCD2 foci formation in MIA PaCa2, PANC-1, Hs 766T, Capan-1 and PL11 cells. Images from one of the representative experiment are shown. **E)** Oxaliplatin sensitivity was assessed in pancreatic cancer cells. **F)** PL5 pancreatic cancer cells were transfected with siRNA oligos against *BRCA2* and *FANCD2*. Quantitative PCR was performed after 48 hours of transfection to confirm the knockdown (inset). A short term (7-days) cell survival assay was performed after treatment with different concentration of MK-1775. The average of three different experiments was shown for each cell line in all the graphs.

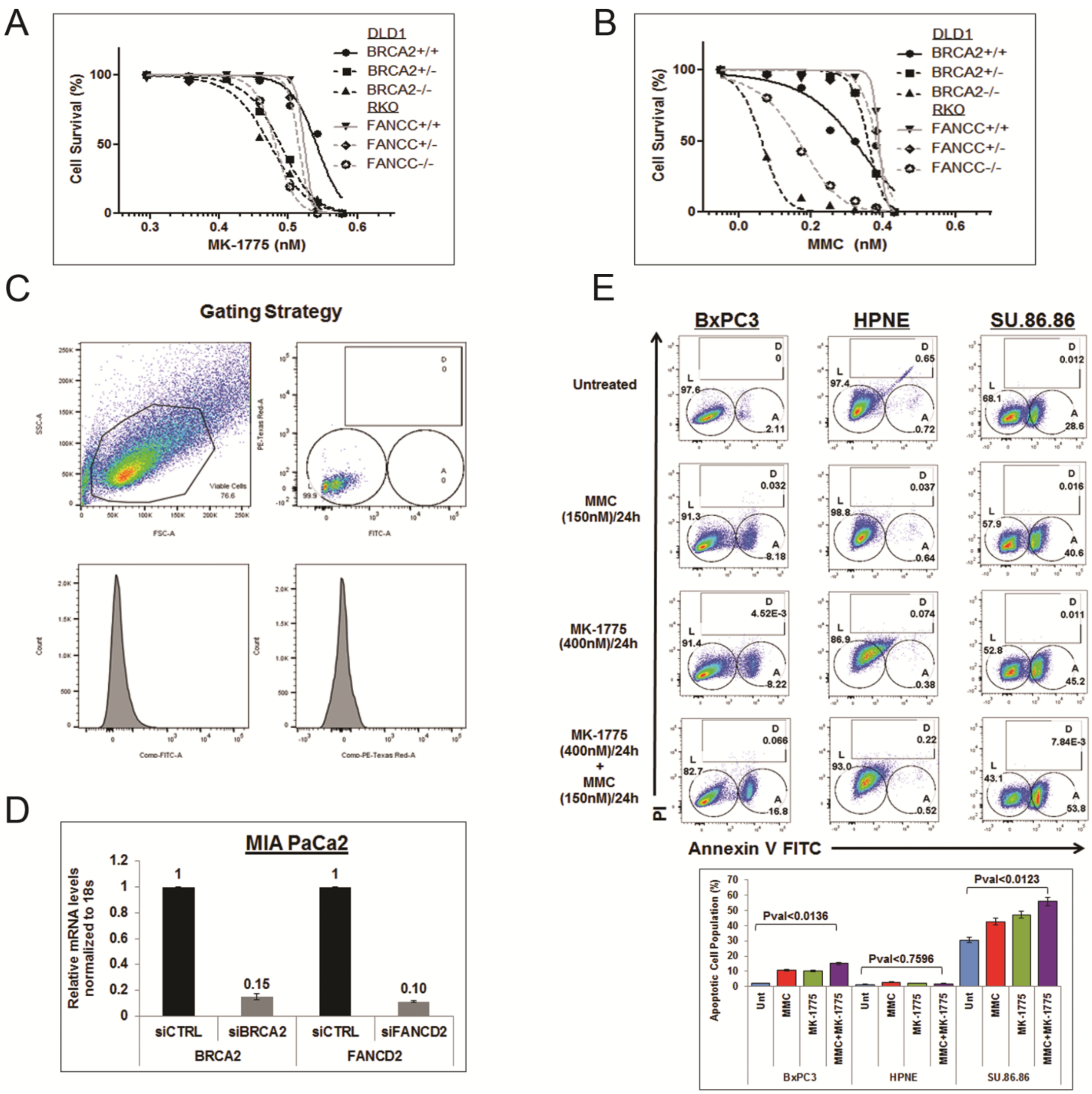

Figure S2: Human adenocarcinoma RKO cell line disrupted in *FANCC* and DLD cell line disrupted in *BRCA2* were treated with **A)** MK-1775 and **B)** MMC and cell viability was assessed after 7 days[1]. **C)** Gating strategy used to analyze the apoptotic cells measured in Figure 2 and Supplementary Figure 2E. **D)** Quantitative PCR was performed after 48 hours of transfection to test the knockdown efficiency of *siBRCA2* and *siFANCD2* in MIA PaCa2 cells to measure the apoptotic cells in Figure 2C-D. **E)** Annexin assay showing the percentage of apoptotic cells in BxPC3, HPNE and SU.86.86 cells upon treatment with MK-1775 and MMC. The graph represents the average of three different experiments for each cell line. L: Live cells; A: Apoptotic cells; D: Dead cells.

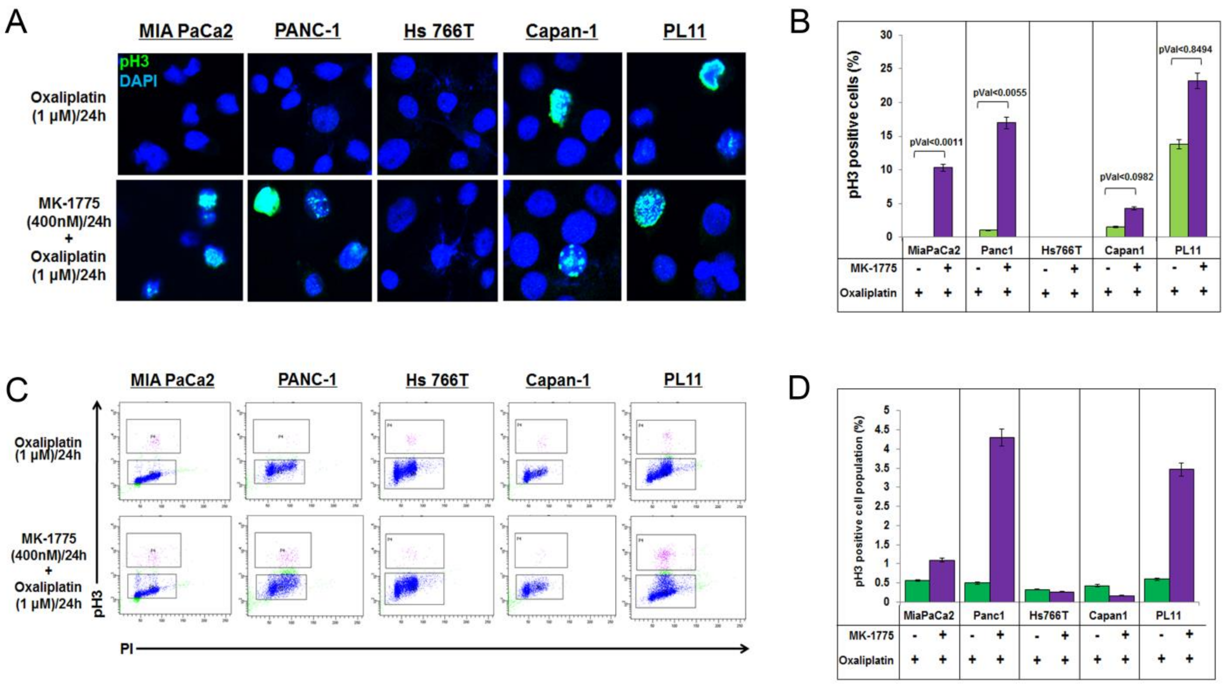

Figure S3: A) MIA PaCa2, PANC-1, Hs 766T, Capan-1 and PL11 cells were treated with MK-1775 and/or oxaliplatin and immunoflorescence was performed. **B)** Quantification of pH3 positive cells in A was plotted. **C)** FACS was performed to determine pH3 positive cells after treatments in MIA PaCa2, PANC-1, Hs 766T, Capan-1 and PL11 cell lines. **D)** Quantification of pH3 positive cells in C was plotted.

Table S1: Table showing IC_{50} values of MK-1775 in PDA and non-PDA cell lines shown in Supplementary Fig. S1A.

Cell Lines	FA-pathway Function	BRCA2 Status	FANCD2 Status	p53 Status	MK-1775 IC_{50} (nM;SE)
**PL5**	Proficient	BRCA2 +/+	FANCC +/+	p53 -/-	285.6±0.046
**Capan-2**	Proficient	BRCA2 +/+	FANCC +/+	p53 -/-	339.0±0.053
**HPNE (non-PDA)**	Proficient	BRCA2 +/+	FANCC +/+	p53 +/+	189.4±0.039

Table S2: Table showing statistical analysis of MK-1775 in PDA cell lines shown in Fig.1A.

	Hs 766T	Capan-1	PL11
**MIA PaCa2**	P value (two-tailed): 0.0004<br>R Square: 0.8088<br>95% confidence interval: 0.2652 to 0.6134	P value (two-tailed): 0.0053<br>R Square: 0.6423<br>95% confidence interval: 0.08125 to 0.3338	P value (two-tailed): 0.0112<br>R Square: 0.5736<br>95% confidence interval: 0.06601 to 0.3783
**PANC-1**	P value (two-tailed): 0.0141<br>R Square: 0.5498<br>95% confidence interval: 0.08473 to 0.5615	P value (two-tailed): 0.0237<br>R Square: 0.4923<br>95% confidence interval: 1.281 to 13.93	P value (two-tailed): 0.0340<br>R Square: 0.4489<br>95% confidence interval: 1.193 to 32.30
**BxPC3**	P value (two-tailed): 0.0338<br>R Square: 0.4499<br>95% confidence interval: -0.2880 to -0.01492	P value (two-tailed): 0.1189<br>R Square: 0.2760<br>95% confidence interval: -0.02575 to 0.1865	P value (two-tailed): 0.1466<br>R Square: 0.2442<br>95% confidence interval: -0.02854 to 0.1600
**SU.86.86**	P value (two-tailed): 0.0083<br>R Square: 0.6021<br>95% confidence interval: -0.6110 to -0.1239	P value (two-tailed): 0.4007<br>R Square: 0.08965<br>95% confidence interval: -0.4881 to 0.2168	P value (two-tailed): 0.3268<br>R Square: 0.1200<br>95% confidence interval: -0.4822 to 0.1816

Table S3: Table showing IC_{50} values of combination treatment of MMC and MK-1775 in PDA cell lines shown in Supplementary Figures S1B and S1C.

Cell Lines	MMC (50 nM); MK-1775 IC_{50} (nM;SE)	MK-1775 (150 nM); MMC IC_{50} (nM;SE)
**MIA PaCa2**	53.35±0.034	9.042±0.029
**PANC-1**	146.1±0.110	21.66±0.034
**Hs 766T**	399.5±0.077	20.19±0.049
**Capan-1**	589.5±0.034	64.04±0.089
**PL11**	270.4±0.091	8.114±0.034
**BxPC3**	594.5±0.147	29.92±0.161
**SU.86.86**	668.8±0.072	151.1±0.100

Table S4: Table showing IC_{50} values of Oxaliplatin in PDA cell lines shown in Supplementary Fig. S1E.

Cell Lines	Oxaliplatin IC_{50} (μM;SE)
**MIA PaCa2**	1.547±0.014
**PANC-1**	0.410±0.056
**Hs 766T**	0.341±0.042
**Capan-1**	0.208±0.016
**PL11**	0.118±0.089

Table S5: Table showing IC_{50} values of MK-1775 in MIA PaCa2 (shown in Fig. 1C), MMC in MIA PaCa2 (shown in Fig. 1D) and MK-1775 in PL5 (shown in Supplementary Fig. S1F) PDA cell line after transfection with *siBRCA2* and *siFANCD2*.

	FA-pathway Function	MIA PaCa2 MK-1775 IC_{50} (nM;SE)	MIA PaCa2 MMC IC_{50} (nM;SE)	PL5 MK-1775 IC_{50} (nM;SE)
**siCTRL**	Proficient	75.6±0.022	70.0±0.079	266.5±0.011
**siBRCA2**	Deficient	326.7±0.087	31.1±0.022	621.4±0.010
**siFANCD2**	Deficient	895.9±0.083	41.6±0.010	496.8±0.024

Table S6: Table showing IC_{50} values of MK-1775 and MMC in genetically disrupted *FANCC* in human adenocarcinoma RKO and *BRCA2* disrupted in DLD cell lines (shown in Supplementary Fig. S2A-B).

Cell Lines	FA-pathway Function	Status	p53 Status	MK-1775 IC_{50} (nM;SE)	MMC IC_{50} (nM;SE)
**DLD1**	Proficient	BRCA2 parent	p53 +/+	2965±0.040	123.1±0.010
**A9.A2**	Deficient	BRCA2 het +/-	p53 +/+	1204±0.013	192.2±0.011
**A10.A3**	Deficient	BRCA2 null -/-	p53 +/+	943.3±0.035	14.46±0.019
**RKO**	Proficient	FANCC parent	p53 +/+	2150±0.014	278.7±0.040
**FCH3C12 alt end +/-/- Cre**	Deficient	FANCC het +/-/-	p53 +/+	1879±0.018	256.1±0.031
**FCE11C12 alt end -/-/- Cre**	Deficient	FANCC null -/-/-	p53 +/+	1069±0.003	29.37±0.022

Table S7: Table showing p-values of Annexin assay shown in Fig. 2A, 2B and S2E.

Cell Lines	MK-1775 vs MMC + MK-1775
**MIA PaCa2**	0.000704375
**PANC-1**	0.00057769
**Hs 766T**	0.124005824
**Capan-1**	0.284367584
**PL11**	0.837069492
**BxPC3**	0.04424436
**SU.86.86**	0.050203006
**HPNE**	0.927587942
